# Concentration Polarization in Ultrafiltration/Nanofiltration for the Recovery of Polyphenols from Winery Wastewaters

**DOI:** 10.3390/membranes8030046

**Published:** 2018-07-21

**Authors:** Alexandre Giacobbo, Andréa Moura Bernardes, Maria João Filipe Rosa, Maria Norberta de Pinho

**Affiliations:** 1 Post-Graduation Program in Mining, Metallurgical and Materials Engineering (PPGE3M), Federal University of Rio Grande Do Sul (UFRGS), Av. Bento Gonçalves 9500, Porto Alegre 91.509-900, Brazil; alexandre_giacobbo@yahoo.com.br (A.G.); amb@ufrgs.br (A.M.B.); 2 Chemical Engineering Department, Instituto Superior Técnico (IST), University of Lisbon, Av. Rovisco Pais, 1049-001 Lisbon, Portugal; 3 Urban Water Division, Hydraulics and Environment Department, National Civil Engineering Laboratory, Av. Brasil 101, 1700-066 Lisbon, Portugal; mjrosa@lnec.pt

**Keywords:** concentration polarization, ultrafiltration, nanofiltration, winery wastewater, polyphenols recovery

## Abstract

Concentration polarization is intrinsically associated with the selective character of membranes and often means flux decline and which causes a subsequent decrease of ultrafiltration and nanofiltration performance. More important is the fact that it acts as a precursor of membrane fouling and creates severe fouling problems in the longer times range. The quantification of its dependence on the operating parameters of cross-flow velocities and transmembrane pressures makes recourse to the film theory to introduce mass-transfer coefficients that generally are calculated by dimensionless correlations of the Sherwood number as a function of the Reynolds and Schmidt numbers. In the present work, the mass-transfer coefficients are obtained through the fitting of experimental results by the pressure variation method. The ultrafiltration/nanofiltration of the winery wastewaters from the racking operation is carried out with the membranes ETNA 01PP (Alfa Laval) and NF 270 (Dow Filmtec) under a wide range of cross-flow velocities and transmembrane pressures up to 15 bar.

## 1. Introduction

The development in the early sixties of the Loeb and Sourirajan asymmetric membranes made possible to envisage the implementation at the industrial scale of the membrane pressure–driven processes, namely ultrafiltration (UF), nanofiltration (NF), and reverse osmosis (RO). However, the high flux characteristics of these membranes and the great capacity of the convective fluxes to bring retained solutes to the membrane surface resulted in solute material accumulation at the membrane/feed interface, as these fluxes are not balanced by the ones of the back diffusion to the bulk feed. This phenomenon designated by concentration polarization (CP) often means flux decline and decrease of UF/NF/RO performance, and more important of all is the fact that CP acts as a precursor of membrane fouling, creating severe fouling problems in the longer times range. In the present work, the consequences of CP on the use of UF/NF for the recovery of valuable compounds from winery wastewaters will be analyzed.

In 2016, the worldwide wine production reached 267 million hectolitres [1], pointing out the wine industry as one of the major agro-industrial activities throughout the world. However, like any agro-industrial process, the generation and treatment of wastewater is a matter of concern. According to Pirra [2], around 0.3–3 L of wastewater is generated per liter of wine produced. These wastewaters are originated from different washing operations during the crushing and pressing of grapes as well as during the racking periods. Their treatment by biological processes, aerobic or anaerobic, encounter severe problems due to the presence of phytotoxic and antibacterial phenolic substances that hinder the biological degradation [3]. On the other hand, many researchers [4,5,6] have shown that phenolic compounds have a high antioxidant activity, along with anti-inflammatory, antiviral, and antimicrobial properties, being flavonoids, flavanols, flavonols, phenolic acids, and stilbenes the main phenolic compounds found in winery wastewaters. Therefore, the recovery of these substances from the wastewaters meets the two-fold objective: biodegradability improvement and byproduct valorisation. In this sense, membrane technologies have been an object of growing interest for separating, concentrating, and purifying the bioactive compounds (polyphenols) from winery wastewaters, UF and NF being the best suited operations for this purpose [7,8,9]. According to the literature [7,8,9], in a cascade-membrane process, microfiltration (MF) may be used to remove suspended solids, colloids, and other impurities, UF to fractionate polyphenols and polysaccharides, and NF to concentrate anthocyanins and other polyphenols.

Nonetheless, even with the technological advances over membrane module design and fluid management, a strong decrease of membrane performance occurs along the system operation. According to Chaabane et al. [10], adsorption, attachment, or accumulation of solutes onto the membrane surface and/or inside membrane pores can occur, causing permeation flux decay along the processing time. Thus, due to the decrease in membrane permeability, the applied feed pressure needs to be increased to maintain the desired flux, resulting in higher energy consumption and operating costs. Besides, cleaning procedures become more frequent, reducing the membrane lifetime. That is, the productivity can be reduced by diverse phenomena as concentration polarization, gel formation, or the different fouling mechanisms: cake formation, solute adsorption, and pore blocking [11]. These phenomena are most of the time the result of UF/NF operation in adverse conditions of concentration polarization. Therefore, the understanding and prediction of this complex phenomenon are key points to optimize pressure-driven membrane operations. 

In a previous work of Giacobbo et al. [12], winery wastewater from the second racking operation was microfiltered with a 0.4 µm pore size membrane to remove the suspended solids and the colloidal matter, yielding a permeate that is object of our concern in the present work. This permeate is a complex mixture mainly composed of polyphenols, sugars, organic acids, and minerals. Therefore, the current work investigates the ultrafiltration and the nanofiltration of this microfiltration permeate, evaluating the effect of operating conditions, cross-flow feed velocity, and transmembrane pressure, on the quantification of concentration polarization through the film theory and recourse to mass-transfer coefficients obtained for the specific experimental conditions of feed circulation and selective permeation.

## 2. Theory

The circulation of a pressurized feed solution of solute A adjacently to a selective UF/NF membrane results in the total or partial solute rejection by the membrane. The rejected solute accumulates near the membrane surface and its concentration, *C*_Am_, is higher than the solute concentration in the bulk feed solution, *C*_Ab_. An intrinsic rejection coefficient, $f_{A}^{\prime }$, is then defined as

(1) $$f_{A}^{\prime }=\frac{C_{\mathrm{Am}}-C_{\mathrm{AP}}}{C_{\mathrm{Am}}},$$

and the apparent rejection coefficient is

(2) $$f_{A}=\frac{C_{\mathrm{Ab}}-C_{\mathrm{AP}}}{C_{\mathrm{Ab}}}.$$

They are related by

(3) fA=fA′fA′+(1−fA′) exp(JPDAWδ)

where *C*_AP_ is the concentration of solute A in the permeate stream, *D*_AW_ is the diffusivity of solute A in water at the boundary layer of thickness *δ*, and *J*_P_ is the membrane permeation flux.

**Figure 1:**
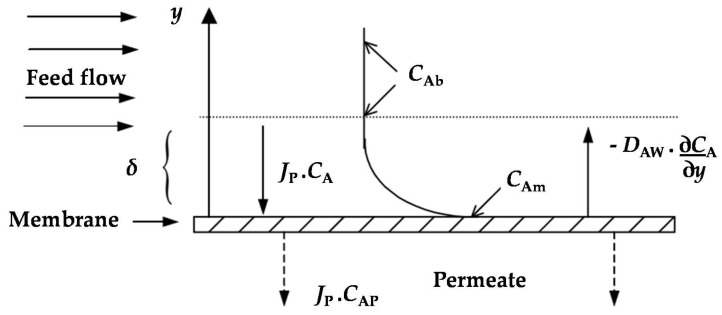
Concentration profile at the boundary layer adjacent to the membrane surface.

The difference between the two rejection coefficients is due to the development of a concentration profile in the laminar boundary layer of thickness *δ*, as schematically shown in Figure 1. A steady state differential mass balance of the convective/diffusive contributions in this boundary layer leads to the quantification of the concentration polarization through Equation (4) [13,14].

(4) CAm−CAPCAb−CAP=eJPDAWδ.

The film theory [15] predicts the mass-transfer coefficient, *k*, as

(5) $$k=\frac{D_{\mathrm{AW}}}{\delta },$$

and substituting Equation (5) in Equation (4), gives

(6) $$\frac{C_{\mathrm{Am}}-C_{\mathrm{AP}}}{C_{\mathrm{Ab}}-C_{\mathrm{AP}}}=e^{\frac{J_{P}}{k}}.$$

Equation (6) describes the concentration polarization making recourse to a mass-transfer coefficient that depends on the feed hydrodynamics, system geometry, and feed physical-chemical properties. The mass-transfer coefficients are most of the times obtained through empirical correlations of the type:

(7) $$Sh=\frac{k\times d_{h}}{D_{\mathrm{AW}}}=a\times Re^{b}\times Sc^{c},$$

where *Sh*, *Re*, and *Sc* are the Sherwood, Reynolds, and Schmidt numbers, *d*_h_ is the hydraulic diameter, and *a*, *b*, and *c* are empirical coefficients that depend on the system under study.

Rosa [16] carried out a very extensive review of the mass-transfer correlations, *Sh* = *Sh* (*Re*, *Sc*), used in UF and NF and introduced a dimensionless hydraulic permeability, $L_{P}^{+}$, to take into account the fact that the membrane is a permeable interface with effect on the concentration profiles developed from the interface to the bulk feed. Using the tangential velocity variation method and the pressure variation method, this author developed the following correlations:

i. For UF,

(8) $$Sh=4.68\times 10^{3}\times Re^{0.50}\times Sc^{0.53}\times L_{P}^{+0.39},$$

with

$$\begin{array}{c} 4706<Re<21,361,\\ 7357<Sc<13,517,\\ 1.67\times 10^{-12}<L_{P}^{+}<1.66\times 10^{-11}; \end{array}$$

ii. And for UF/NF,

(9) $$Sh=1.5\times 10^{4}\times Re^{0.47}\times Sc^{0.33}\times L_{P}^{+0.35},$$

with

$$\begin{array}{c} 2662<Re<21,072,\\ 557<Sc<13,517,\\ 7.75\times 10^{-14}<L_{P}^{+}<1.66\times 10^{-11}. \end{array}$$

In the dimensionless hydraulic permeability, $L_{P}^{+}=\frac{L_{P}}{R_{m}},$ *L*_P_ is the hydraulic permeability (expressed in length units) and *R*_m_ is the membrane radius [16,17]. The tangential velocity variation method requires the assumption of a Reynolds number exponent, *b*, that Rosa [16] assumed to be the Grober exponent of 0.5, characteristic of a turbulent flow under development [18].

In the present work, the mass-transfer coefficients are obtained through the fitting of experimental data obtained in a permeation testing cell where the fluid dynamics have the same characteristics of a turbulent flow under development as in the work of Rosa [16]. For the use of the pressure variation method [16], the Equation (3) is rearranged in the form:

(10) $$\ln\left(\frac{1-f_{A}}{f_{A}}\right)=\ln\left(\frac{1-f_{A}^{\prime }}{f_{A}^{\prime }}\right)+\frac{1}{k}\times J_{P}.$$

The linear regression of the experimental values of $\ln\left(\frac{1-f_{A}}{f_{A}}\right)$ vs. $J_{P}$ yields a straight line whose slope is 1/*k* and the intercept at the origin, $\ln\left(\frac{1-f_{A}^{\prime }}{f_{A}^{\prime }}\right)$, allows the determination of $f_{A}^{\prime }$.

## 3. Materials and Methods

### 3.1. Winery Wastewater

Wastewater generated in the second racking from red wine production was collected in a winery located in the Vale dos Vinhedos Region in Brazil. In a previous study [12], this wastewater was microfiltered with a 0.4 µm pore size membrane to yield a permeate that is used as feed solution to the UF/NF experimental runs conducted in the present work.

For this wastewater characterization, total organic carbon (TOC) was measured in a TOC-LCPH carbon analyzer (Shimadzu Scientific Instruments Inc., Kyoto, Japan), total polysaccharide content was determined by the phenol-sulfuric acid method and expressed as mg L^−1^ of glucose [19], and monomeric anthocyanins were analyzed according to pH differential method [20] and the results expressed in mg L^−1^ of malvidin-3-glucoside (Mv3g). The determination of total polyphenols content was carried out by a colorimetric method using a UV-Vis spectrophotometer (PG Instruments, Lutterworth, UK), by measuring the absorbance at 280 nm [21,22] and expressing the results as mg L^−1^ of gallic acid equivalent (GAE). In the present work, the studies were restricted to the total polyphenols content.

The composition of the wastewater used as feed solution is displayed in Table 1.

### 3.2. Membranes 

Experiments were conducted with two commercial membranes: (i) ETNA 01PP, a composite fluoro polymer membrane from Alfa Laval, Nakskov, Denmark, with molecular weight cut-off (MWCO) of 1000 Da (according to the supplier) and (ii) NF 270, a polypiperazine membrane from DOW-Filmtec, Edina, MN, USA, with a 300 Da MWCO. For the NF 270 membrane, the apparent rejection coefficients to sodium chloride and sodium sulfate are 45.9% and 99.4%, respectively, and for the ETNA 01PP membrane they are 12.8% and 67.2%, respectively [23].

### 3.3. UF/NF Permeation Experiments

Permeation runs were carried out in laboratory flat-cell units with 14.5 cm^2^ of membrane surface area, described in previous works [8,24]. Firstly, the membranes were compacted by circulating deionized water (conductivity < 2 µS cm^−1^) pressurized at 10 bar for 3 h. This avoids pressure effects on the membrane structure in the subsequent experiments. The pure water permeation fluxes, *J*_w_, were determined at transmembrane pressures, *ΔP*, varying from 3 to 10 bar. The slope of the straight line *J*_w_ vs. *ΔP* yields the membrane hydraulic permeability, *L*_P_. Permeation experiments with the feed solution were performed in total recirculation mode, characterized by the streams (retentate and permeate) being continuously recirculated to the feed tank. These experiments were conducted to assess the variation of the permeation fluxes and rejections to polyphenols as a function of transmembrane pressure (3–15 bar) and cross-flow feed velocities (CFV) of 0.48, 0.72, and 0.96 m s^−1^. Between runs, membranes were washed with deionized water until the hydraulic permeability achieved at least 90% of the original value.

Feed temperature was set at 25 ± 1 °C and the stabilization time for each run was 30 min. After that, permeate samples were collected for analysis. Feed concentration was determined by the average of two sample collections: one at the beginning and another at the end of each run.

## 4. Results and Discussion

### 4.1. UF/NF Permeation Experiments

The hydraulic permeability (*L*_P_) of the NF 270 membrane is 8.7 kg h^−1^ m^−2^ bar^−1^ and the one of the ETNA 01PP is 8.8 kg h^−1^ m^−2^ bar^−1^.

**Figure 2:**
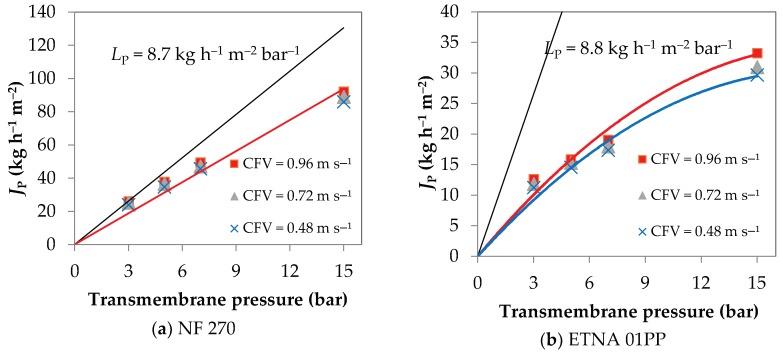
Permeation fluxes (*J*_P_) as a function of transmembrane pressure at three cross-flow feed velocities: (**a**) NF 270 membrane; (**b**) ETNA 01PP membrane.

Figure 2 displays the permeation flux as a function of the transmembrane pressure for the NF 270 (Figure 2a) and ETNA 01PP (Figure 2b) membranes. Three cross-flow feed velocities are considered.

The NF 270 permeation fluxes increase linearly with transmembrane pressure for all the three cross-flow feed velocities, meaning that, even at the lowest CFV the shear rate was high enough to maintain the concentration polarization layer sufficiently small to the point that it did not restrict the permeation flux. Nonetheless, the slope of the straight line of *J*_P_ vs. transmembrane pressure is 6.3 kg h^−1^ m^−2^ bar^−1^ and is significantly lower than the one corresponding to the hydraulic permeability.

The ETNA 01PP membrane shows typical characteristics of UF permeation fluxes. At low pressures, up to approximately 2 bar, the permeation fluxes increase linearly with the transmembrane pressure with a slope of 6.6 kg h^−1^ m^−2^ bar^−1^ for all cross-flow velocities. Above 2 bar there is a deviation from linearity, i.e., the critical flux is reached [25], and the curves of *J*_P_ vs. transmembrane pressure present much lower permeation fluxes when compared to the ones of pure water. The critical flux is typically defined as the flux leading to a first deviation from the linearity of flux with transmembrane pressure and, theoretically, below it there is virtually no fouling occurrence, while above it fouling is observed [25,26]. Indeed, working at CFV of 0.48 and 0.72 m s^−1^, the ETNA 01PP membrane does not yield substantial difference in permeation fluxes, while at 0.96 m s^−1^ higher permeation fluxes were obtained. The permeate fluxes decline is more pronounced in the lower CFV and in the higher transmembrane pressures and such conditions favor the occurrence of concentration polarization phenomenon, a precursor of fouling. Conversely, the critical flux had a slight increase with CFV showing its dependence with the hydrodynamics, which is in agreement with other works [27,28]. In this sense, according to Bacchin et al. [25], the critical flux is a criterion for the transition between concentration polarization and fouling.

**Figure 3:**
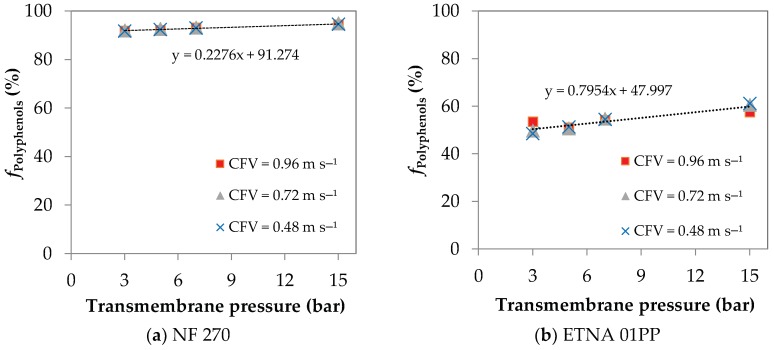
Variation of polyphenols rejection with the transmembrane pressure at three cross-flow feed velocities: (**a**) NF 270 membrane; (**b**) ETNA 01PP membrane.

The apparent rejection coefficients to polyphenols, *f*_Polyphenols_, as a function of the transmembrane pressure are shown in Figure 3a for the NF 270 membrane and in Figure 3b for the ETNA 01PP membrane. The results correspond to experiments at three different CFV. For both membranes, the apparent rejection coefficients to polyphenols increase with the transmembrane pressure and are practically independent of the CFV. According to Wijmans and Baker [29], by the solution-diffusion model, the pure water flux (or more generally the pure solvent flux) increases with transmembrane pressure, while the solute flux is independent of it. Consequently, the apparent rejection coefficient increases with the transmembrane pressure. Besides that, at higher transmembrane pressures the concentration polarization is more intense, leading to the formation of a more selective layer onto the membrane surface. This behaviour is more pronounced for the ETNA 01PP membrane that has lower rejection coefficients to polyphenols and higher permeation fluxes decline (Figure 2). The straight line fitting *f*_Polyphenols_ vs. transmembrane pressure has a slope 3.5 times higher for the ETNA 01PP than for the NF 270 membrane.

### 4.2. Concentration Polarization Quantification

**Figure 4:**
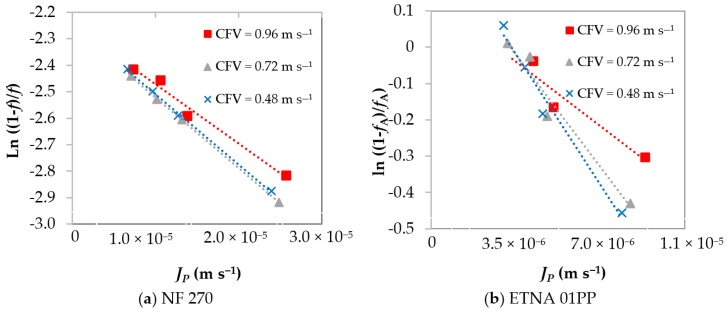
Variation of apparent rejection coefficients with the transmembrane pressure at three cross-flow feed velocities: (**a**) NF 270 membrane; (**b**) ETNA 01PP membrane.

Considering the polyphenols as the solute A and as earlier stated, the representation of the permeation results in the form of $\ln\left(\frac{1-f_{A}}{f_{A}}\right)$ vs. $J_{P}$ (Figure 4) yields a straight line whose slope is the inverse of the mass-transfer coefficient, 1/*k*, and the intercept at the origin allows predicting the intrinsic rejection coefficient, $f_{A}^{\prime }$.

Table 2 presents the intrinsic rejection coefficients, $f_{A}^{\prime }$, and the experimental mass-transfer coefficients, *k*, for both membranes at different operating conditions. For both membranes, the increase in CFV leads to an increase in the *k* value. The values of $f_{A}^{\prime }$ are around 0.9 for the NF 270 and around 0.4 for the ETNA 01PP membrane. These results are in line with those achieved through the Rosa correlations, Equations (8) and (9), for the mass-transfer coefficients that increase with CFV (Table 3). In fact, the mass-transfer coefficients calculated through the Rosa correlations are of the same order of magnitude although slightly lower or slightly higher than the ones of the present work for the NF 270 membrane or ETNA 01PP membrane, respectively.

The mass-transfer coefficients obtained in this work are slightly superior to the ones reported on the literature when UF or NF were applied to concentrate solutions-containing polyphenols [30,31]. For instance, Todisco et al [31] achieved an experimental *k* of 0.95 × 10^−5^ m s^−1^ by using a 40 kDa UF membrane at a CFV of 3.2 m s^−1^ (*Re* = 33,000) during the clarification of a black tea containing 1970 mg L^−1^ polyphenols. Dammak et al [30] found an experimental *k* of 0.88 × 10^−5^ m s^−1^ employing a 250 Da NF membrane to concentrate solutions with 300–2700 mg L^−1^ oleuropein. In these studies, the polyphenols content in the feed solution is one or two orders of magnitude higher than the one evaluated at the present work, and this justifies the *k* values slightly lower than the ones we found (Table 2 and Table 3).

On the other hand, the results obtained for polyphenols must have been influenced by the water background organic and inorganic matrices. Indeed, as displayed in Table 1, the winery wastewater under study is a complex mixture that, besides polyphenols, contains polysaccharides, organic acids, and minerals. As a result, the other macromolecules also play an important role in mass transfer during the filtration process. In addition to increasing the solution viscosity, they may interact with polyphenols by enhancing concentration polarization and rejection coefficients. It is expected that a synthetic-binary solution having the same polyphenols content evaluated here (~27 mg L^−1^) would present higher mass-transfer coefficients and lower polyphenols rejection than the ones reported in the present work.

The differences reported on the *k* values as well as the effect of the water matrix demonstrate the need to use case-specific mass transfer correlations for an accurate prediction of the concentration-polarization phenomenon.

The concentration polarization with regard to the accumulation in the boundary layer of the rejected polyphenols (designated by solute A) is quantified through Equation (6) and the results are shown in Table 4 for NF 270 and ETNA 01PP membranes at varying conditions of cross-flow velocities and transmembrane pressures. 

The concentration polarization increases with the increase of the transmembrane pressure and with adverse hydrodynamic conditions of low cross-flow velocities.

## 5. Conclusions

The concentration polarization is calculated with recourse to mass-transfer coefficients that, in most of the situations, are obtained through literature correlations not adequate to the geometry and hydrodynamics of the ultrafiltration/nanofiltration permeation units. In the present work they were obtained experimentally and compared to the ones obtained by correlations for permeation units with similar hydrodynamics of turbulent flow under development and considering a permeable membrane/feed interface. This analysis highlights the importance of using case-specific mass transfer correlations when accurate predictions of the concentration-polarization phenomenon are envisaged to enable an efficient design and operation of membrane systems for resource recovery.

## Figures and Tables

**Table 1:** Physical-chemical characteristics of the feed solution.

Parameter	Feed Solution
TOC (mg L^−1^ C)	716 ± 10.8
Turbidity (NTU)	<1.0
Conductivity (µS cm^−1^)	241 ± 2.0
Total Polysaccharides (mg L^−1^ Glucose)	10.1 ± 0.4
Total Polyphenols (mg L^−1^ GAE)	26.6 ± 0.1
Monomeric Anthocyanins (mg L^−1^ Mv3g)	4.20 ± 0.1

**Table 2:** Intrinsic rejection coefficient and experimental mass-transfer coefficient for NF 270 and ETNA 01PP membranes at different cross-flow feed velocities.

CFV (m s^−1^)	NF 270		ETNA 01PP	
	$f_{A}^{\prime }$	*k* × 10^−5^ (m s^−1^)	$f_{A}^{\prime }$	*k* × 10^−5^ (m s^−1^)
0.48	0.90	3.77	0.42	1.02
0.72	0.91	3.75	0.43	1.18
0.96	0.90	4.47	0.47	2.06

**Table 3:** Mass-transfer coefficients obtained through the Rosa correlations, *k*, for NF 270 (Equation (9)) and ETNA 01PP (Equation (8)) membranes at different cross-flow feed velocities.

CFV (m s^−1^)	*k* × 10^−5^ (m s^−1^)	
	NF 270	ETNA 01PP
0.48	2.92	1.63
0.72	3.54	2.00
0.96	4.05	2.31

**Table 4:** Assessment of concentration polarization by phenolic compounds at different cross flow velocities (CFV) and transmembrane pressure (*ΔP*) for NF 270 and ETNA 01PP membranes.

CFV (m s^−1^)	*ΔP* (bar)	NF 270			ETNA 01PP		
		*C*_AP_ (mg L^−1^)	*C*_Am_ (mg L^−1^)	$\left(\frac{C_{Am}-C_{AP}}{C_{Ab}-C_{AP}}\right)$	*C*_AP_ (mg L^−1^)	*C*_Am_ (mg L^−1^)	$\left(\frac{C_{Am}-C_{AP}}{C_{Ab}-C_{AP}}\right)$
0.48	3.0	2.18	31.30	1.19	13.68	31.22	1.36
	5.0	2.02	33.73	1.29	12.92	33.25	1.49
	7.0	1.85	36.49	1.40	12.07	35.39	1.61
	15	1.42	48.93	1.89	10.30	46.92	2.25
0.72	3.0	2.13	31.62	1.21	13.35	30.81	1.32
	5.0	1.96	34.23	1.31	13.11	32.41	1.43
	7.0	1.83	36.95	1.42	12.02	34.25	1.53
	15	1.36	50.23	1.94	10.46	43.86	2.07
0.96	3.0	2.18	30.87	1.18	12.34	29.22	1.19
	5.0	2.10	33.08	1.27	13.02	29.81	1.24
	7.0	1.85	35.50	1.36	12.18	30.80	1.30
	15	1.50	46.01	1.78	11.28	35.26	1.57
